# Development of a Method to Determine the Environmental Burden of Diseases and an Application to Identify Factors Driving Changes in the Number of PM_2.5_-Related Deaths in China between 2000 and 2010

**DOI:** 10.1021/envhealth.4c00048

**Published:** 2024-06-12

**Authors:** Ning Kang, Pengfei Li, Tao Xue, Tong Zhu

**Affiliations:** † Institute of Reproductive and Child Health, National Health Commission Key Laboratory of Reproductive Health/Department of Epidemiology and Biostatistics, Ministry of Education Key Laboratory of Epidemiology of Major Diseases (PKU), School of Public Health, Peking University Health Science Center, Beijing 100191, China; ‡ Institute of Medical Technology, Peking University Health Science Center, Beijing 100191, China; § Advanced Institute of Information Technology, Peking University, Hangzhou 311215, China; ∥ State Environmental Protection Key Laboratory of Atmospheric Exposure, and Health Risk Management and Center for Environment and Health, Peking University, Beijing 100871, China; ⊥ State Key Joint Laboratory of Environment Simulation and Pollution Control, College of Environmental Sciences and Engineering, Peking University, Beijing 100871, China

**Keywords:** health burden, decomposition analysis, air pollution, fine particulate matter, premature deaths

## Abstract

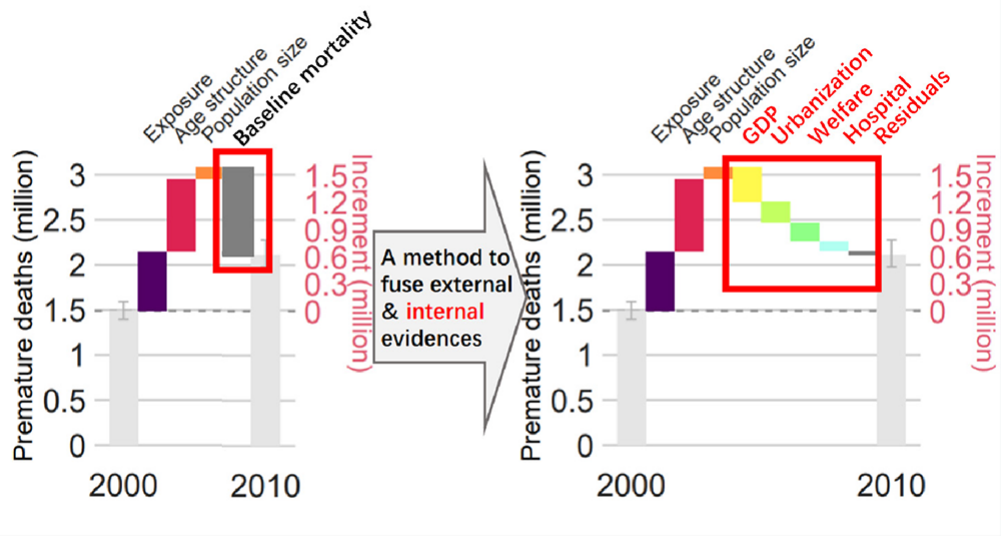

The attributable burden is codetermined by the exposure level and nontarget characteristics. However, the conventional method of health impact assessment based on preestablished exposure–response functions includes only a few well-known characteristics and thus is insufficient to capture the comprehensive variation. We aimed to develop a method to fuse health impact assessment with epidemiological analysis and to identify factors driving baseline risk. The method was applied to identify the factors underlying the change in the number of fine particulate matter (PM_2.5_) related deaths in China between 2000 and 2010. During the study period, the number of PM_2.5_-related deaths across mainland China increased by 0.62 (95% CI: 0.57, 0.69) million, with 0.65 (95% CI: 0.47, 0.91) million, 0.55 (95% CI: 0.39, 0.79) million, and 0.11 (95% CI: 0.06, 0.18) million deaths being associated with increased PM_2.5_ exposure, population aging, and growth in population size, respectively. However, economic growth, urbanization, improvement of welfare services, and improvement of hospital services resulted in 0.25 (95% CI: 0.15, 0.40) million, 0.16 (95% CI: 0.10, 0.27) million, 0.16 (95% CI: 0.09, 0.26) million, and 0.09 (95% CI: 0.05, 0.15) million fewer deaths, respectively. Results indicated that increased exposure was the major driver of the change in the number of PM_2.5_-related deaths, and economic growth was the main driver of increased resilience to air pollution.

## 1 Introduction

Global environmental changes, including climate change, air pollution, suboptimal temperatures, and natural hazards, are major risk factors for poor health.^1^ To understand how environmental stressors, attributable risk—the amount of disease that can be attributed to a specific risk factor—is a critical metric.^2^ Identifying the drivers of attributable risk is crucial for addressing the public health challenges posed by global environmental changes, and it can inform more targeted, effective, and evidence-based strategies to protect human health. For instance, population aging has been identified as an additional factor to increase the mortality burden attributable to air pollution and climate changes.^3,4^ However, existing methods used to calculate the attributable risk of the target factor do not distinguish the health effects of multiple nontarget factors, making it difficult to decompose the factors driving poor health.

Taking air pollution as an example, ambient exposure to fine particulate matter (PM_2.5_) has been linked to many diseases and contributes to a number of premature deaths worldwide.^5,6^ Many previous studies have shown that PM_2.5_-related deaths are codetermined by the exposure level, age structure, population size, and baseline mortality rate.^7−10^ However, the baseline mortality rate is ambiguous. Previous studies typically described changes in the baseline mortality rate in terms of improvements or worsening of healthcare,^7^ medical status,^10^ or socioeconomic status without any estimation,^11,12^ which might mislead readers. According to the classical method used to calculate premature deaths attributable to PM_2.5_ (see eq 1), the baseline mortality rate reflects total mortality, which is affected by not only nontarget factors (e.g., unhealthy behaviors) but also target factors (PM_2.5_ in the present study). Because the baseline mortality rate reflects all factors, it should be derived directly from surveys or registries, observation-based estimates (e.g., interpolated estimates), or state-of-the-art estimates based on the best data available, such as the global burden of disease (GBD) estimates. To better understand the drivers of PM_2.5_-related deaths, a novel method of risk attribution taking into account coexposure of nontarget factors (e.g., age structure, population size, urbanization, and others) is needed.

The term “attributable risk” is used in both health impact assessments (e.g., GBD assessments) and epidemiological investigations, particularly observational studies, regardless of whether the causal inference method is applied.^13−15^ Health impact assessments of target factor rely on an evidence-based exposure–response relationship, which is usually derived from information independent of nontarget factors.^16−18^ The exposure–response relationships are usually obtained from two methods. First, many scholars (e.g., David Hume, who views causation only as an inference drawn from associations) and organizations (e.g., the United States Environmental Protection Agency) infer causality based on the weight of evidence from various studies, and the attributable risk is interpreted in terms of attribution of causes.^19^ In contrast, epidemiological investigations, which aim to derive an exposure–response association or relative risk from observational data, must also report the attributable risk for accurate interpretation of the estimated association.^20,21^ For example, the STROBE guidelines for reporting epidemiological results state the following: “If relevant, consider translating estimates of relative risk into absolute risk for a meaningful time period.”^22^ However, some researchers do not recommend reporting the absolute risk to avoid overinterpreting an association as a causal relationship in an observational epidemiology.^23^ This study does not aim to determine whether or not the attributable risk should be interpreted in terms of the attribution of causes. We view attributable risk as a useful health metric, similar to the relative risk and hazard ratio, and we recommend that it always be reported to aid interpretation of the results.^20^ We distinguish between two types of attributable risk: *externally attributable risk* for health impact assessment and *internally attributable risk* for an epidemiological investigation. The externally attributable risk is based on a generalizable exposure–response relationship with external validity; in contrast, the internally attributable risk is based on an empirical exposure–response association that may be valid within the analyzed sample or target population.

For a specific exposure, either externally or internally attributable risk should be selected at the design stage according to data availability and the study aim. In brief, externally attributable risk applies to well-known risk factors. Both health effects and their distributions must be obtained from observational data (e.g., surveys or censuses) or state-of-the-art estimates. Internal attributable risk depends on an epidemiological investigation, which in turn depends not only on the availability of observational data but also on the properties of the data set. For instance, when a key confounder is omitted, conducting an epidemiological study can be problematic, and the observational data should not be utilized to generate the internal attributable risk.^24^ Because the two methods are suitable for exposures with different features, combining them to develop a novel modeling framework incorporating multiple coexposures together with the target exposure is of interest. Even for the burden of disease attributable to a single exposure, coexposures (e.g., socioeconomic conditions and behavioral risk factors) can affect the spatiotemporal distribution of the attributable risk.

In this study, we first show that a model fusing externally and internally attributable risks is equivalent to an epidemiological model incorporating the effects of well-known risk factors as prior knowledge or offset information. Next, we apply our novel method to data from our previous study, which showed the comparability of internally and externally attributable risks in terms of PM_2.5_ exposure. To illustrate the value of our novel method, we apply it to understand the forces driving the change in the number of premature deaths in China attributable to PM_2.5_ between 2000 and 2010.

## 2 Methods

### 2.1 Externally Attributable Risk

External risk factors were well-known risk factors, and each exposure–response was defined by previous studies (e.g., GBD estimates). In the present study, PM_2.5_ was defined as the external risk factor, and we used the Global Exposure Mortality Model (GEMM) to quantify premature deaths attributable to PM_2.5_ exposure at the county level in China.^17^ Let us first consider the classical method used to calculate the attributable number (AN) of premature deaths in a health impact assessment:
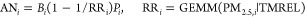
1where *i* denotes the spatiotemporal unit of health impact assessment (county–year for this study), PM_2.5,*i*_ denotes the exposure level, TMREL denotes the theoretical minimum risk exposure level (directly using the GEMM default value in this study), RR_*i*_ denotes the relative risk of PM_2.5_ exposure, *P*_*i*_ denotes the population size, and *B*_*i*_ denotes the observed all-cause mortality rate. Under ideal conditions, as in this study, we can collect relevant observation data from surveys or censuses. However, for most previous studies, *B*_*i*_ was estimated or interpolated. Specifying how the estimation procedure affects the validity of the attributable risk is beyond the scope of this study and will thus be discussed only briefly in the following sections.

Equation 1 can be modified to incorporate multiple risk factors. Let us redefine the observed all-cause mortality rate as *B*_*i*_ = *B*_0_ × RR_*i*_ × RR′_*i*_ and input it into eq 1:
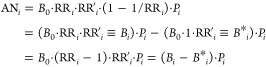
2where *B*_0_ denotes the reference mortality rate, usually corresponding to the minimum among global observations, RR′_*i*_ denotes the relative risk of nontarget risk factors, and B*_i_ denotes the mortality rate under the counterfactual scenario of PM_2.5_ removal.

We can further extend nontarget risk factors in eq 2 to incorporate additional well-known risk factors (i.e., external risk factors) and unidentified risk factors at the county level. Let us convert eq 2 into
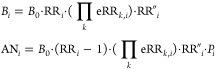
3where eRR denotes the relative risk of a coexposure obtained from well-established sources, and RR″_*i*_ denotes the relative risk of unidentified risk factors.

### 2.2 Internally Attributable Risk

For the internal risk factors, such as medical services and economic status, the defined exposure–response functions are lacking. In this situation, it is necessary to derive an empirical relative risk via an epidemiological investigation by using data from the present study and then to estimate the internally attributable risk. Assuming that PM_2.5_ is empirically associated with the outcome, let us consider the following additive nonlinear regression:
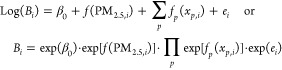
4where *x*_*p*_ denotes the *p*th covariates empirically associated with the target health metric of interest, *f*_*p*_ denotes the corresponding nonlinear function (nature cubic spline with four knots in the present study), and *e*_*i*_ denotes the residuals. Based on the fitness of eq 4, we calculated the internally attributable risk for PM_2.5_ exposure as follows:
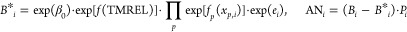
5Given the following definitions:
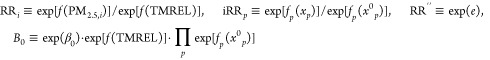
Equation 5 can be transformed as follows:
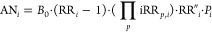
6where *x*^0^ denotes the reference value for each covariate. The value of *x*^0^ will not change the internally attributable risk for PM_2.5_ but determines *B*_0_. Equations 3 and 6 differ only in the source of risk.

### 2.3 Attributable Risk Derived via the Fusion Method

Combining eqs 3 and 6 is a straightforward way to calculate the attributable risk:Step 1:
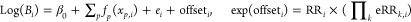
Step 2:
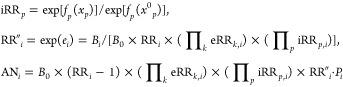
7where “offset” denotes the prior knowledge used to explain the variation in outcomes based on well-known risk factors. Step 1 is an updated version of the regression model considering the offset, and the inputs in step 2 (i.e., *B*_0_ and RR″_*i*_) are parametrized according to eq 3. In the above method, step 1 is only slightly different from eq 4, because it estimates the exposure–response functions for empirical risk factors (iRR) after adjusting for the impact of both the target exposure (RR) and well-known coexposures (eRR) by introducing the offset term. Offset-adjusted models, as in step 1, have been widely utilized to model the association between environmental exposure and population-level disease statistics, such as the mortality rate or cancer registry rate. Because the two-step method combines iRR and eRR, it can be said to fuse internally and externally attributable risks.

In our example analysis, the target exposure is PM_2.5_, and the RR is given by the GEMM. Age structure is another well-known risk factor, and the eRR is given by a survival function based on the reference life table developed by GBD researchers. The empirical covariates are indicators of economic status, welfare services, hospital services, and urban residence, and the corresponding iRRs are estimated from a county-level regression analysis.

### 2.4 Environmental and Population Data

The demographic and environmental data in this study are the same as those in our previous study, the details of which have been published in that study.^15^ Briefly, county-level population, urbanization, and mortality data were derived from two census data sets (2000 and 2010), related publications, and the website of the National Bureau of Statistics of China (http://www.stats.gov.cn/). After excluding Hong Kong, Macau, Taiwan, and some islands in the South China Sea, a total of 2836 county-level geographic units were included in the present study. The population was divided into age groups from 0 to 84 years old in 5-year intervals and a group aged 85 years or older. The annual concentrations of PM_2.5_ were derived from a well-constructed data set provided by van Donkelaar et al.,^25^ with a spatial resolution of 0.1° × 0.1°. Population-weighted county-level PM_2.5_ concentrations were derived according to the number of included pixels. Other socioeconomic variables, including the gross domestic product, number of hospital beds, and number of welfare home (e.g., nursing homes for the elderly or orphans) beds, were collected from various editions of the China Urban Statistical Yearbook and China County Statistical Yearbook. The variables were further normalized on a per capita basis.

### 2.5 Epidemiological Analysis

We applied eq 7 to analyze county-level mortality data. As mentioned before, we performed a log–linear regression analysis with fixed effects:
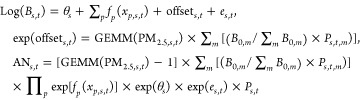
8where *s*, *t*, and *m* denote indexes for county, year, and age group, respectively; *x*_*p*_ denotes covariates; and *B*_0,*m*_ denotes the ideal mortality rate derived from the reference life table. The offset term captures the external risk factors: PM_2.5_ and age . The covariates were gross regional product per capita, number of hospital beds per capita, number of welfare beds per capita, and the percentage of urban residents.

To further identify the drivers of temporal changes in the number of PM_2.5_-related deaths, we calculated the difference between two attributable risks (one based on the 2010 value and the other based on the 2000 value). Given that setting rest factors at the 2000 or 2010 level could affect the calculations, we considered all possible combinations and obtained the distribution of the difference between the two attributable risks. In this study, we considered two external factors, PM_2.5_ exposure level and age structure , as well as four internal factors (*x*_*p*_).

All analyses were performed by using R software (R Development Core Team, Vienna, Austria). Uncertainty ranges were obtained from a Monte Carlo simulation. A simple R-demo has been added in the Supporting Information.

## 3 Results

### 3.1 Externally Attributable Risk of PM_2.5_-Related Mortality

Let us first recall some key findings from our previous study. We calculated the PM_2.5_-related all-cause mortality using several preestablished exposure–response functions, including the GEMM (for all-cause mortality), GEMM-5COD (the GEMM for five cause-specific mortality), and an integrated exposure–response (IER) model. We also derived an empirical association between the PM_2.5_ concentration and mortality rate from the same census data analyzed in this study. Among the three preestablished functions, the GEMM generated the externally attributable risk that agreed best with the internally attributable risk estimated from the empirical association. As discussed previously, the empirical association might be limited due to the omission of key confounders, such as the smoking rate. Therefore, this study used the GEMM to reestimate the externally attributable risk according to eq 1 or 2. The exposure levels and baseline mortality rate were also presented in our previous study and are thus not repeated here.

**Figure 1:**
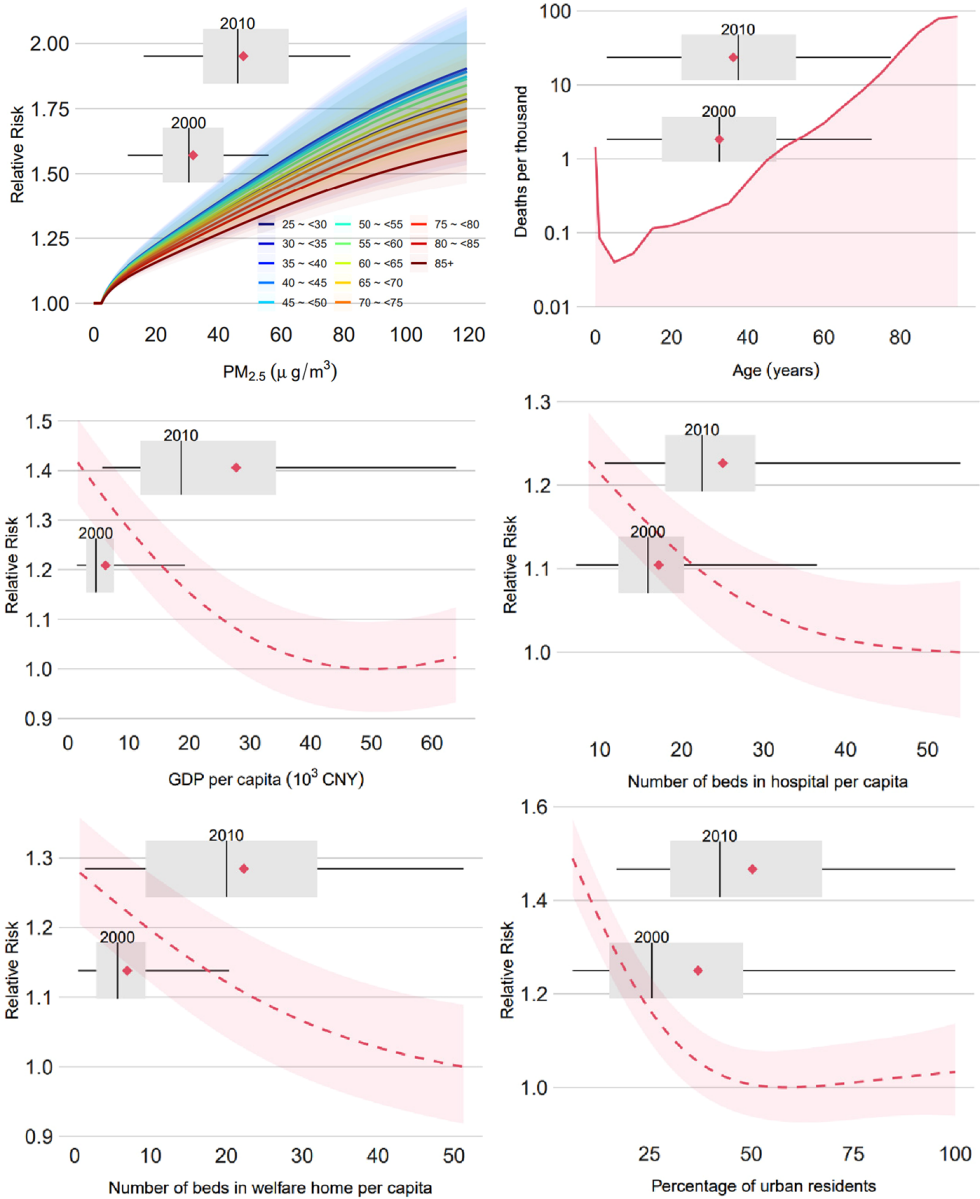
Exposure–response functions of fine particulate matter (PM_2.5_) and coexposures with all-cause mortality rate in China. The solid lines denote preestablished functions, the dashed lines denote the empirical functions estimated directly from a census-based epidemiological study, the boxplots denote the distributions of risk factors, and the red points denote the mean value. GDP, gross domestic product.

**Figure 2:**
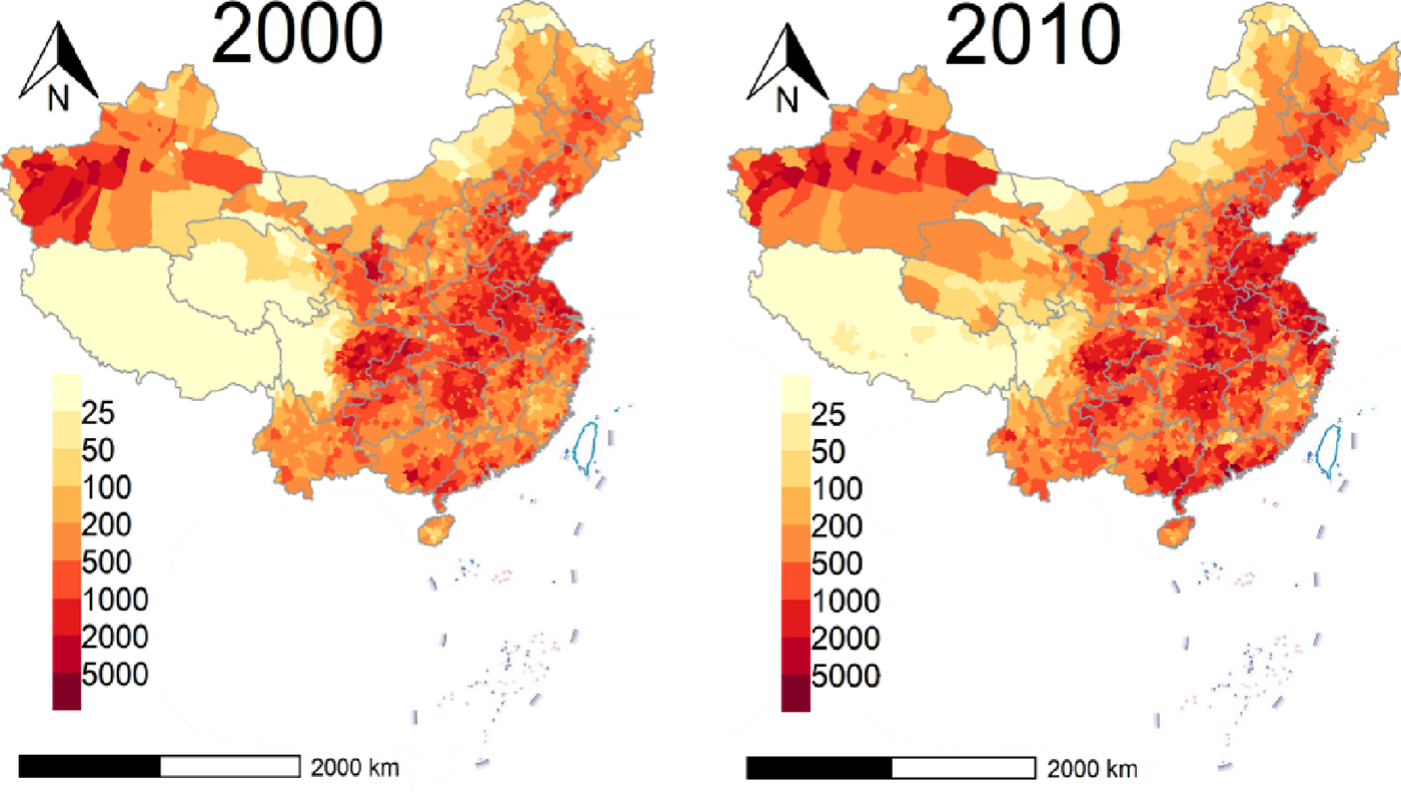
Numbers of premature deaths attributable to PM_2.5_ exposure in China at the county level according to the global exposure mortality model.

The statistical distributions of PM_2.5_ and its coexposures are shown in Figure 1, and the spatial distributions of PM_2.5_-related deaths estimated by the GEMM are shown in Figure 2. Based on our reanalysis, there were 1.49 (95% confidence interval [CI]: 1.40, 1.59) million and 2.11 (95% CI: 1.98, 2.28) million premature deaths attributable to PM_2.5_ exposure in 2000 and 2010, respectively. During the study period, the number of PM_2.5_-related deaths increased by 0.62 (95% CI: 0.57, 0.69) million, comparable with a previous result based on the GEMM model (0.61 [95% CI: 0.56, 0.66] million); the slight difference is due to a difference in the baseline mortality rate. The spatial distributions showed the regions in China where the health impact of PM_2.5_ has been greatest. The externally attributable risk was consistent with other studies. The hotspots were in regions such as Beijing and its surrounding area, the Yangtze River Delta, and the Fenwei Plain. Additionally, some counties in Xinjiang also had high PM_2.5_ attributable mortality, which may be partially caused by the high concentration of PM_2.5_ from dust sources.

### 3.2 Empirical Associations of Coexposures

After controlling for the externally attributable risks of PM_2.5_ exposure and aging (solid lines in Figure 1), we further analyzed the associations between the all-cause mortality rate and the coexposures at the county level. All four indicators, which consistently improved from 2000 to 2010 with socioeconomic development, were negatively associated with mortality, as shown by the dashed lines in Figure 1. The exposure–response functions for the number of welfare home beds and number of hospital beds were homogeneous and nonlinear. For gross domestic product and urbanization, the functions indicated a potentially protective effect of high-level exposure.

### 3.3 Drivers of Changes in the PM_2.5_-Related Mortality Rate from 2000 to 2010

**Figure 3:**
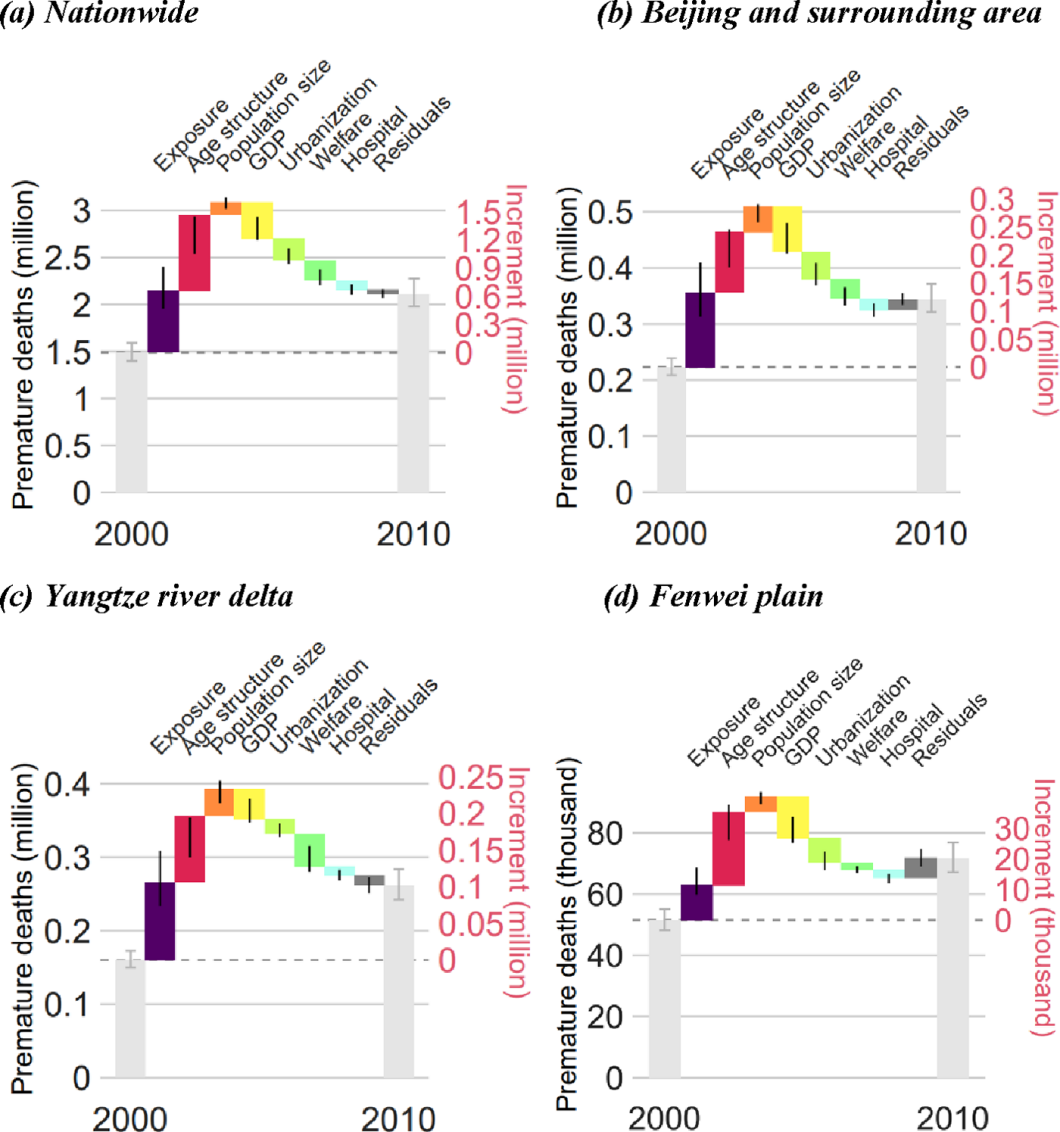
Drivers affecting the PM_2.5_-related mortality rate from 2000 to 2010 across China or the key subregions. (a) Nationwide, (b) Beijing and surrounding area, (c) Yangtze River Delta, and (d) Fenwei Plain. GDP, gross domestic product.

Figure 3 shows the factors affecting the number of PM_2.5_-related deaths between 2000 and 2010. During the study period, the number of PM_2.5_-related deaths increased by 0.62 (95% CI: 0.57, 0.69) million, with 0.65 (95% CI: 0.47, 0.91) million, 0.55 (95% CI: 0.39, 0.79) million, and 0.11 (95% CI: 0.06, 0.18) million deaths being associated with increased PM_2.5_ exposure, population aging, and growth in population size, respectively. In contrast, economic growth, urbanization, improvement of welfare services, and improvement of hospital services resulted in 0.25 (95% CI: 0.15, 0.40) million, 0.16 (95% CI: 0.10, 0.27) million, 0.16 (95% CI: 0.09, 0.26) million, and 0.09 (95% CI: 0.05, 0.15) million fewer deaths, respectively. All other factors made only a small contribution to the decrement in PM_2.5_-related deaths (0.03 [95% CI: −0.01, 0.09] million). Our results suggest that the major factor promoting resilience to air pollution is economic growth, followed by urbanization and improved welfare. In contrast, population aging makes the population more vulnerable to air pollution, and it was the second most important contributor to the increased number of PM_2.5_-related deaths.

The main drivers of PM_2.5_-related deaths differed among regions (Figure 3c,d). During 2000–2010, increased exposure was the major driver of the increase in PM_2.5_-related deaths in most areas. In regions where the population was aging particularly rapidly, such as the Fenwei Plain, increased vulnerability was caused mainly by the change in age structure. Improved welfare services, which mainly target the elderly, were the most important protective factor in the Fenwei Plain, supplanting economic growth.

## 4 Discussion

The attributable burden of PM_2.5_ is codetermined by the exposure level and other factors. To better understand the driving forces behind the changes in the health burden of PM_2.5_, the present study developed a novel method that fuses the well-established exposure–response functions with epidemiology analyses. By using this method, results indicated that economic growth, improved welfare services, improved hospital services, and urbanization could slow the growth of PM_2.5_-related deaths between 2000 and 2010 across mainland China. The study highlights the need for more advanced methods, such as the one developed here, to conduct a deeper investigation into the drivers of environmental disease burden.

**Figure 4:**
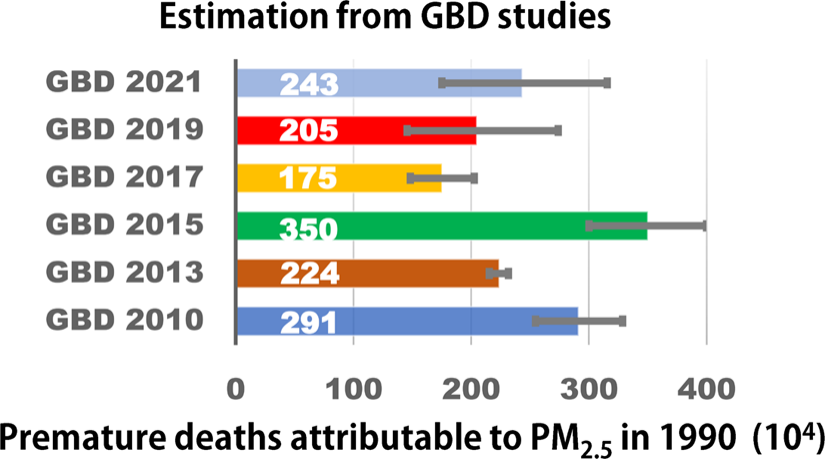
Premature deaths attributable to PM_2.5_ exposure in 1990 estimated by different methods based on externally attributable risk. GBD, global burden of diseases.^50−54^

Many studies have estimated the number of premature deaths caused by PM_2.5_ exposure based on preestablished exposure–response functions, including the IER, GEMM, and meta-regression-Bayesian, regularized, trimmed (MR-BRT). However, the results vary among functions. For example, our estimates for the same spatiotemporal domain using different GBD assessments varied considerably (Figure 4), similar to previous studies focusing on China. PM_2.5_-related deaths estimated by the IER model in 2010 ranged from 1.24 million to 1.36 million among studies.^26,27^ The corresponding value in 2015 varied from 1.82 million^28^ to 1.94 million^29^ in different GEMM-based studies, and it ranged from 0.95 million^12^ to 1.34 million^18^ in studies using the MR-BRT function. This variation might be explained by differences in population, mortality, or exposure data among studies. In general, GEMM-based estimates for China were higher than those based on the IER model^17,29^ or the MR-BRT model.^30^ Additionally, few studies have evaluated PM_2.5_-related deaths using the internally attributable risk. In a nationwide cohort study, Li et al. estimated that 1.77 million people aged >65 years experienced premature mortality due to PM_2.5_ exposure in 2010.^31^ Another nationwide cohort study estimated that PM_2.5_ exposure contributed to 2.68 million deaths in 2015, accounting for 27.4% of all deaths.^32^ However, except for our previous study,^33^ no studies have compared the externally and internally attributable risks for PM_2.5_ exposure. In the future, a novel method combining externally and internally attributable risks, just like ours, will be useful for determining whether incorporating a preestablished exposure–response function into an epidemiological investigation provides insights into the comparability of the two types of attributable risks. Such a comparative analysis is beyond the scope of this study and will be left to future works.

Although drivers of PM_2.5_-related deaths have also been reported in previous studies, they differed according to the baseline mortality interpretation. Previous studies attributed changes in the baseline mortality rate in terms of improved healthcare,^7^ economic development,^12^ or the combined effects of urbanization, income, demographics, employment, age distribution, and healthcare access.^11,12^ The baseline mortality rate is not spatiotemporally invariant and depends on many factors. In previous studies of externally attributable risk, the method used to calculate baseline mortality rate was not elucidated in sufficient detail. As mentioned previously, the baseline mortality rate reflects all risk factors and should be correlated with the exposure level. Therefore, the findings of previous studies, including ours, might have been mis- or overinterpreted. In most studies, baseline mortality rates were usually derived from reanalysis of observational databases provided by authoritative organizations (e.g., the GBD database),^34,35^ surveys, registries, yearbooks, or censuses, or from surveillance data.^13,14,32,36,37^ However, such data usually have a coarse resolution (e.g., national- or provincial-level data). Some studies calculated the number of deaths in subregions (e.g., at the county or pixel level), hypothesizing that mortality rates are invariant across subregions.^9,29,38^ Other studies assumed that mortality depended on exposure and calculated the baseline mortality rate by adjusting the nationwide average according to local exposure data.^11,14,39^ In contrast, previous studies on internally attributable risk focused only on the target exposure and thus underestimated the importance of coexposure of nontarget factors. Therefore, few studies have distinguished drivers of PM_2.5_-related deaths based on internally attributable risks. Observational data should be representative of a large, or even the entire, population; high data quality should be sacrificed if necessary to achieve a large sample size. However, for several reasons, a large, moderate-quality data set is not always suitable to develop exposure–response functions for the target exposure. First, confounders may be omitted, and large studies are more likely to have systematic biases or large amounts of missing data, even for key variables.^40^ Second, exposure misclassification may occur. Exposures in large-population studies is always derived from model estimates or approximations rather than precise measurements.^41^ Third, ecological bias, i.e., the modifiable areal unit problem, may be present because individual-level records are aggregated by geographical units in large-population studies.

The following limitations of this study should be addressed in future studies. First, the number of coexposures was limited. For instance, lifestyle factors are considered important for mortality^18^ but were not considered in the present study because such data were not available. However, as the number of residuals was small, the results can still be considered reliable. Second, the assumption of homogeneity made for the preestablished exposure–response function may be invalid. The health effects of PM_2.5_ depend on its composition, and theoretically, the dose–response relationship varies regionally and temporally. Third, the assumption that the effects of different risk factors are additive may also be invalid. For instance, GDP, urbanization, and welfare services are correlated with each other,^42^ which could give rise to multicollinearity; this issue merits further attention. Fourth, we only used GEMM to quantify premature deaths attributable to PM_2.5_, which may not work well for the Chinese population given different pollution levels and characteristics, climates, population behaviors, and demographics. However, considering that the GEMM model covered much of the global exposure distribution (including the evidence from China),^17^ the estimation of GEMM may be still convincing. Finally, this study tested our method only at the population level. For individual-level microsimulation studies, the approach taken to incorporate internally and externally attributable risks should be slightly modified.^43^

## 5 Conclusion

This study presents a novel method combining health impact assessments based on well-established exposure–response functions with epidemiological analyses to explore the associations between the baseline mortality rate and coexposures of interest. The method maximizes the number of explanatory factors used to estimate the burden of diseases attributable to environmental exposures. We demonstrated that economic growth, improved welfare services, improved hospital services, and urbanization could slow the growth of PM_2.5_-related deaths between 2000 and 2010 across mainland China. Our findings demonstrate the importance of adaption and resilience to prevent premature deaths attributable to air pollution.

## Data Availability

The data analyzed during the current study are available from the corresponding author on reasonable request.

## Supporting Information Available

A simple demo code (PDF)
Demo data (XLSX)
