# Supplementary material for: Development of a Method to Determine the Environmental Burden of Diseases and an Application to Identify Factors Driving Changes in the Number of PM2.5-Related Deaths in China between 2000 and 2010
Source: Environ Health (Wash). 2024 Jun 12;2(9):642–50. doi: 10.1021/envhealth.4c00048 (PMC11540115; doi:10.1021/envhealth.4c00048)
Supplement: Supplementary file 1 — eh4c00048_si_001.pdf [file eh4c00048_si_001.pdf]

Simple Demo For
Development of a method to determine the environmental burden of diseases and an application to identify factors driving changes in the number of PM2.5-related deaths in China between 2000 and 2010

Ning Kang, Pengfei Li, Tao Xue, Tong Zhu

2024-5-10

In order to make our approach more intuitive, it is repeated here in the simplest case possible. **It is important to note that all data are randomly changed and the results do not represent anything**.

1. Generating data

Here, `GB` is the city, `dC` is the death count,`u2t~Care` are non-target risk factors, `PM` is the target risk factor, `R0` is the sum of *reference mortality rate * age-group population*, `wave` is the survey time, `RR` is the risk ratio of `PM` calculated by `GEMM`, and `RR = R1/R0`.

```
library(tidyverse)
library(gnm)
library(mgcv)
library(gam)
# load("dom_data.RData")
head(dom_data)
```

```
##   GB       dC       u2t       GDP       Med       Care        PM
R1
## 0  1 3749.280  99.92136 11000.164 23.24807 11.236919 57.50000 1586.2
63
## 1  2 4660.005  93.08084 10042.994 20.94090  7.839223 57.00000 2050.3
71
## 2  3 2105.562  94.83509 11250.246 17.98226  9.836285 58.00000 1020.7
31
## 3  4 3514.151  95.87155 11393.669 21.29471  8.122406 59.50000 1516.6
71
## 4  5 9002.192  97.57047  9796.079 25.33614  8.768122 53.40000 4514.9
62
## 5  6 6545.244 106.76705  8964.938 22.33342  4.524063 56.33333 2668.0
35
##          R0 wave       RR
## 0 1115.7719 2000 1.421673
## 1 1447.6035 2000 1.416390
## 2  699.0075 2000 1.460258
## 3 1041.7888 2000 1.455834
## 4 3178.0016 2000 1.420692
## 5 1869.7910 2000 1.426916
```

2. Externally attributable risk

Externally attributable risk means the **exposure-response was derived from well-established previous study**. This demo regarded the `PM2.5` as the external risk factor, and we defined the RR for PM2.5 was `GEMM(PM)` from previous evidence, as well as age `structure`, `population size`, and `reference mortality`.

```r
# Not show:
# ~~RR is the calculated RR ; GEMM(PM);
# ~~Calculation and data of `age structure` , `population size` and `re
ference mortality` can be found in Method section.
```

3. Internally attributable risk

Internally attributable risk means the **exposure-response was derived from the present study with the evidence from previous study**. This demo regarded the u2t,GDP,Med,u2tas the internal risk factor. `R1` is the external risk including the *PM2.5,age structure,population size*, and *reference mortality*. Then the `residual` of model is the **unidentified risk factor**.

```r
m <- gnm(log(dC)~GDP+Care+Med+u2t+offset(log(R1)),data=dom_data,elimina
te = GB)
summary(m)

##
## Call:
## gnm(formula = log(dC) ~ GDP + Care + Med + u2t + offset(log(R1)),
##     eliminate = GB, data = dom_data)
##
## Deviance Residuals:
##     Min       1Q   Median       3Q      Max
## -5.4781  -0.1377   0.0000   0.1377   5.4781
##
## Coefficients of interest:
##        Estimate Std. Error t value Pr(>|t|)
## GDP  -1.746e-06  5.412e-07  -3.226  0.00127 **
## Care -8.924e-03  7.007e-04 -12.736  < 2e-16 ***
## Med  -3.871e-03  1.207e-03  -3.207  0.00136 **
## u2t  -8.225e-03  8.172e-04 -10.064  < 2e-16 ***
## ---
## Signif. codes:  0 '***' 0.001 '**' 0.01 '*' 0.05 '.' 0.1 ' ' 1
##
## (Dispersion parameter for gaussian family taken to be 0.1902548)
##
## Residual deviance: 527.01 on 2770 degrees of freedom
## AIC: 8242.7
##
## Number of iterations: 2

res=matrix(m$y-m$fitted.values,ncol=2) # unmeasured risk  in two stages
```

4. Calculation the PM2.5 attributable risk

~stage2000:

```r
# Our methods:
demo_data_2000 <- dom_data[dom_data$wave==2000,]
demo_data_2000$R1 <- demo_data_2000$R1
predict_pm25 <- predict(m,newdata=demo_data_2000 ,se.fit = F)+res[,1]

demo_data_2000$R1 <- demo_data_2000$R0
predict_nonpm25 <- predict(m,newdata=demo_data_2000 ,se.fit = F)+res[,
1]
sum(exp(predict_pm25)-exp(predict_nonpm25) ,na.rm = T)

## [1] 1485096

# Trditional methods:
demo_data_2000$AF=(demo_data_2000$RR-1)/demo_data_2000$RR
sum(demo_data_2000$AF*demo_data_2000$dC)

## [1] 1485096

Burden_2000=sum(exp(predict_pm25)-exp(predict_nonpm25) ,na.rm = T)
```

~stage2010:

```r
# Our methods:
demo_data_2010 <- dom_data[dom_data$wave==2010,]
demo_data_2010$R1 <- demo_data_2010$R1
predict_pm25 <- predict(m,newdata=demo_data_2010 ,se.fit = F)+res[,2]

demo_data_2010$R1 <- demo_data_2010$R0
predict_nonpm25 <- predict(m,newdata=demo_data_2010 ,se.fit = F)+res[,
2]
sum(exp(predict_pm25)-exp(predict_nonpm25) ,na.rm = T)

## [1] 2113669

# Trditional methods:
demo_data_2010$AF=(demo_data_2010$RR-1)/demo_data_2010$RR
sum(demo_data_2010$AF*demo_data_2010$dC)

## [1] 2113669

Burden_2010=sum(demo_data_2010$AF*demo_data_2010$dC)
```

~change

```r
total_DR = Burden_2010 - Burden_2000
total_DR

## [1] 628572.4
```

5. Decomposition of drivers

Similar with previous study, decomposition of drivers should calculate the all scenarios. **Here we only show one scenario**. For example, let us change the GDP.

```
head(demo_data_2010)

##        GB        dC       u2t      GDP      Med     Care   PM
R1
## 01      1  3646.530 100.72377 31834.06 27.74410 36.60591 84.0 1551.27
70
## 11000  2  4845.783  92.34754 30388.73 31.87753 36.68983 82.0 1897.12
68
## 2838   3  2529.530 101.79547 32516.89 33.22678 34.02250 87.5  951.97
73
## 3100   4  3030.560  97.55657 31790.30 30.83014 34.86285 86.0 1502.89
94
## 4100   5 14625.537  98.67030 34026.24 31.05347 35.87000 81.7 6073.57
37
## 5100   6  9696.334  94.61390 27248.45 26.43196 27.74227 80.5 3878.29
79
##              R0 wave       RR        AF
## 01    1551.2770 2010 1.567645 0.3621006
## 11000 1897.1268 2010 1.560700 0.3592618
## 2838   951.9773 2010 1.579916 0.3670551
## 3100  1502.8994 2010 1.581865 0.3678347
## 4100  6073.5737 2010 1.567130 0.3618909
## 5100  3878.2979 2010 1.570229 0.3631501

head(demo_data_2000)

##   GB       dC       u2t       GDP      Med      Care        PM
 R1
## 0   1 3749.280  99.92136 11000.164 23.24807 11.236919 57.50000 1115.7
719
## 1   2 4660.005  93.08084 10042.994 20.94090  7.839223 57.00000 1447.6
035
## 2   3 2105.562  94.83509 11250.246 17.98226  9.836285 58.00000  699.0
075
## 3   4 3514.151  95.87155 11393.669 21.29471  8.122406 59.50000 1041.7
888
## 4   5 9002.192  97.57047  9796.079 25.33614  8.768122 53.40000 3178.0
016
## 5   6 6545.244 106.76705  8964.938 22.33342  4.524063 56.33333 1869.7
910
##          R0 wave       RR        AF
## 0 1115.7719 2000 1.421673 0.2966035
## 1 1447.6035 2000 1.416390 0.2939798
## 2  699.0075 2000 1.460258 0.3151894
## 3 1041.7888 2000 1.455834 0.3131084
```

```
## 4 3178.0016 2000 1.420692 0.2961178
## 5 1869.7910 2000 1.426916 0.2991880
```

```r
tmp <- demo_data_2000
tmp$GDP <- demo_data_2010$GDP

change <- sum(exp(predict(m,newdata=tmp ,se.fit = F)+res[,1]) - exp(pre
dict(m,newdata=demo_data_2000 ,se.fit = F)+res[,1]))
change
```

```
## [1] -191466.6
```
